# Impact of body mass index on long-term blood pressure variability: a cross-sectional study in a cohort of Chinese adults

**DOI:** 10.1186/s12889-018-6083-4

**Published:** 2018-10-22

**Authors:** Haojia Chen, Ruiying Zhang, Qiongbing Zheng, Xiuzhu Yan, Shouling Wu, Youren Chen

**Affiliations:** 1 Shantou University Medical College, Shantou, Guangdong China; 2 Department of Cardiology, Second Affiliated Hospital of Shantou University Medical College, Shantou, Guangdong China; 3 Department of Cardiology, Kailuan Hospital, North China University of Science and Technology, Tangshan, China; 4 Graduate School, North China University of Science and Technology, Tangshan, China; 5 School of Foreign Language, Guangdong Polytechnic Normal University, Guangzhou, Guangdong China

**Keywords:** Body mass index, Blood pressure variability, Blood pressure

## Abstract

**Background:**

Obesity and overweight are related to changes in blood pressure, but existing research has mainly focused on the impact of body mass index (BMI) on short-term blood pressure variability (BPV). The study aimed to examine the impact of BMI on long-term BPV.

**Methods:**

Participants in the Kailuan study who attended all five annual physical examinations in 2006, 2008, 2010, 2012, and 2014 were selected as observation subjects. In total, 32,482 cases were included in the statistical analysis. According to the definition of obesity in China, BMI was divided into four groups: underweight (BMI < 18.5 kg/m^2^), normal weight (18.5 ≤ BMI < 24.0 kg/m^2^), overweight (24.0 ≤ BMI < 28.0 kg/m^2^), and obese (BMI ≥ 28.0 kg/m^2^). We used average real variability to evaluate long-term systolic BPV. The average real variability of systolic blood pressure (ARV_SBP_) was calculated as (|sbp2 − sbp1| + |sbp3 − sbp2 | + |sbp4 − sbp3| + |sbp5 − sbp4|)/4. Differences in ARV_SBP_ by BMI group were analyzed using analysis of variance. Stepwise multivariate linear regression and multiple logistic regression analyses were used to assess the impact of BMI on ARV_SBP_.

**Results:**

Participants’ average age was 46.6 ± 11.3 years, 24,502 were men, and 7980 were women. As BMI increases, the mean value of ARVSBP gradually increases. After adjusting for other confounding factors, stepwise multivariate linear regression analysis showed that ARVSBP increased by 0.077 for every one-unit increase in BMI. Multiple logistic regression analysis indicated that being obese or overweight, compared with being normal-weight, were risk factors for an increase in ARVSBP. The corresponding odds ratios of being obese or overweight were 1.23 (1.15–1.37) and 1.10 (1.04–1.15), respectively.

**Conclusions:**

There was a positive correlation between BMI and ARVSBP, with ARVSBP increasing with a rise in BMI. BMI is a risk factor for an increase in ARVSBP.

**Trial registration:**

Registration No.: CHiCTR-TNC1100 1489; Registration Date: June 01, 2006.

## Background

The proportion of people who are obese or overweight continues to increase, with obese adults and children currently accounting for 37% and 24% of the population worldwide, respectively [1]. In 2010, research on the global burden of disease showed that obesity or overweight were responsible for 3.9% of the years of life lost and 3.8% of the disability-adjusted life years and caused a global death toll of 3.4 million [2]. Body mass index (BMI) is an indicator used to systematically measure obesity and overweight status. BMI has been broadly applied in research relating to obesity and overweight because of its convenience of use. Research has shown that high BMI is a risk factor for hypertension and cardiovascular events (CVEs) [3–7]. Hypertension and risk of CVEs are, respectively, 4.17 and 1.46 times higher among those who are obese, compared with those of normal weight [3, 6].

Obesity and overweight are not only risk factors for hypertension; they are also related to changes in blood pressure (BP). Faramawi [8] found that, for every one-unit increase in BMI, short-term blood pressure variability (BPV) increased by 0.25. In addition to short-term BPV, BP also has long-term variability, which is caused by factors including the environment and behaviors [9–11], but existing research on BMI and BPV have mainly focused on the impact of BMI on short-term BPV. To the best of our knowledge, there has been almost no research about the effect of BMI on long-term BPV. The Kailuan study (Registration No.: CHiCTR-TNC1100 1489) examines risk factors and interventions for CVEs based the full population of staff members working for the Kailuan Group. Since 2006, a physical examination has been conducted every 2 years among the observation group. This examination includes measurements of BP, height, and weight, offering an opportunity to study the impact of BMI on long-term BPV.

## Methods

### Study participants

Following a cross-sectional study design, since 2006–2007 (hereafter “2006”), 11 hospitals affiliated with Kailuan Hospital have conducted physical examinations on their current, dismissed, and retired staff members, collecting body measurements and biochemical indexes. All the staff came from China. They were included in the study subjects if they had met the following criteria: aged 18 years or older; with complete data on height and weight; with no history of CVEs. The same medical staff who conducted the first health examination continued to examine the same group of people at the same sites every 2 years in the same chronological order, making up the second (2008–2009), third (2010–2011), fourth (2012–2013), and fifth (2014–2015) waves of data collection (referred to in this article as “2008,” “2010,” “2012,” and “2014,” respectively). The investigation content, anthropometric measurements, and biochemical index tests were identical at each wave. Those who had missed any systolic blood pressure (SBP) test across the five study waves and incidence of CVEs between first and fifth physical examinations were excluded. In accordance with the Helsinki Declaration, this study has been approved by the Ethics Committee of Kailuan General Hospital, and written informed consent was obtained from all individuals in the observation group.

### Data collection

Details of the epidemiological investigation and anthropometry index have been described elsewhere [12]. Smoking was operationalized as smoking at least one cigarette per day on average in the last year. Drinking was operationalized as consuming more than 300 ml of liquor (alcohol concentration > 50% volume per volume) per day for at least 1 year. Physical exercise was defined as exercising more than three times per week, with each time lasting more than 30 min. CVEs included stroke (namely, hemorrhagic stroke or ischemic stroke) and myocardial infarction.Trained medical staff members checked all of the inpatient diagnoses for individuals in the study group at the hospitals in the Kailuan Group and at the hospitals that were municipally listed for medical insurance every year. These staff members also recorded final events. All of these diagnoses were confirmed by professional doctors in line with inpatient records.

For the measurement of BP, subjects were required to refrain from smoking or drinking tea or coffee for 30 min before the measurement and to sit upright for 15 min quietly. The measurement was taken with the subject sitting with his/her bare right upper arm slightly stretched out, with the elbow lying at the same level as the heart. A suitable cuff was selected for the subject’s upper arm circumference and was then wound with moderate tension, close to the skin of the upper arm, with the cuff’s upper edge approximately 2.5 cm above the chelidon and centered over the brachial artery. The first four physical examinations used an adjusted mercury sphygmomanometer to test the BP of the right brachial artery. The SBP reading used Korotkoff sounds phase one, and the DBP reading used the fifth phase. For the fifth physical examination, an Omron electronic sphygmomanometer (HEM-8102A, Omron Co., Ltd. Daling China) was used to measure the BP of the right brachial artery. During each physical examination, BP was tested three times, with an interval of 1 min between tests, and the average of these three readings was recorded as the final BP of each subject.

To measure height and weight, trained medical staff members use an adjusted scale (model RGZ-120) to measure the subject’s height and weight from 7:30 a.m. to 9:00 a.m. For these measurements, the subjects were barefoot, had nothing on their heads, wore light clothing, and stood upright. Height was measured to the nearest 0.1 cm, and weight was measured to the nearest 0.1 kg.

### Biochemical measurements

Blood samples taken from the antecubital vein were collected in EDTA tubes from the participants fasting overnight. By centrifugating at 3000 g for 10 min (centrifuge radius of 17 cm) at room temperature, plasma was then isolated. The measurement of supernatant serum was carried out in 4 h. Hexokinase/glucose-6-phosphate dehydrogenase method was adopted to measure fasting blood glucose. Total cholesterol (TC) was measured enzymatically (Mind Bioengineering Co. Ltd., Shanghai, China). All biochemical variables were measured by an automatic biochemical analyzer (Hitachi 747; Hitachi, Tokyo, Japan).

### Relevant definitions

#### Hypertension and diabetes

Hypertension was defined as having a history of hypertension, SBP ≥ 140 mmHg and/or diastolic blood pressure (DBP) ≥ 90 mmHg, or SBP < 140 mmHg and/or DBP < 90 mmHg but taking antihypertensive drugs. Criteria for diabetes was a fasting glucose level of 126 mg/dL (7.0 mmol/L) or greater, or use of medications used to treat hyperglycemia.

#### BMI

BMI was calculated as weight/height^2^ (unit: kg/m^2^).

#### Long-term blood pressure variability

Measures of long-term BPV [13] include the standard deviation of BP, the coefficient of variation, the variability uncorrelated with mean BP, and the average real variability (ARV). ARV requires that the order of the BP readings be in line with the formula. This measurement can better predict damage to the target organ [14–16]. For this reason, ARV was adopted in the present study to estimate long-term BPV. The average real variability of systolic blood pressure (ARVSBP) was calculated using the following formula: ARVSBP = (|sbp2 − sbp1| + |sbp3 − sbp2| + |sbp4 − sbp3| + |sbp5 − sbp4|)/4, where sbp1, sbp2, sbp3, sbp4, and sbp5 represent the subject’s SBP at the first through the fifth physical examinations.

### Statistical methods

All of the information from the physical examinations was recorded by unifying trained professional staff members and gathered by Kailuan General Hospital. SPSS 13.0 (SPSS, Chicago, IL, USA) was used for the data analysis. Averages ± standard deviations (x̅ ± s) were calculated for continuous variables. Single-factor analysis of variance was used for group comparisons, comparing averages using the least significant difference test in cases of variance homogeneity or Dunnett’s T3 test in cases of variance non-homogeneity For categorical variables, n (%) was reported, and the χ^2^ test was used for group comparisons. Stepwise multiple linear regression models were used to analyze the impact of BMI on ARVSBP, and logistic regression models were used to estimate the risk of an increase in ARVSBP associated with BMI. For the latter analysis, the subjects were divided into two groups according to ARVSBP (less than vs. equal to or greater than the average ARVSBP [12.64]). Differences were considered statistically significant if *P* < 0.05 (two-sided test).

Including patients with hypertension and those taking antihypertensive drugs, and using different methods of measuring BP may affect our assessment of long-term BPV. Therefore, patients with hypertension, those taking antihypertensive drugs, and the fifth physical examination (which used a electronic sphygmomanometer to measure BP) were sequentially excluded in a sensitivity analysis, where we calculated the effect of BMI using partial regression coefficients, odds ratios (OR), and 95% confidence intervals (95% CI) of ARVSBP. Here, differences were considered statistically significant if *P* < 0.05 (two-side test).

## Results

### Study subjects’ general information

**Fig. 1:**
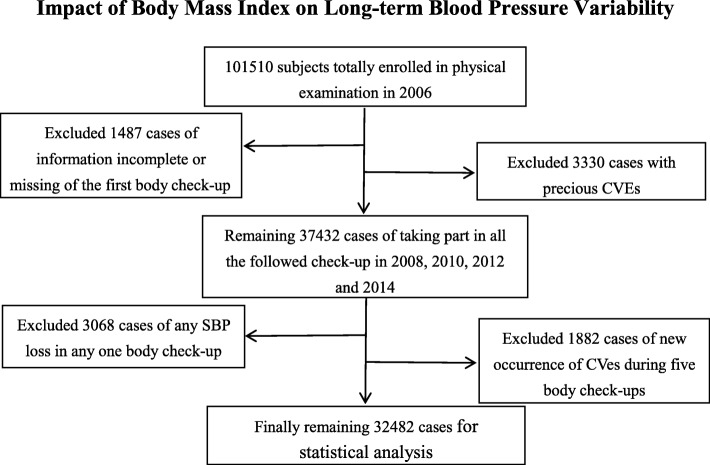
Inclusion/exclusion flowchart for study subjects

A total of 101,510 subjects were enrolled in the physical examination in 2006. Excluding the 3330 cases with previous CVEs and the 1487 cases with incomplete information or missing weight or height at the first physical examination reduced the sample to 37,432 cases who took part in physical examinations in all of the study years (2008, 2010, 2012, and 2014). We then excluded the 3068 cases with missing data on SBP at any physical examination and the 1882 cases of new occurrence of CVEs during the study period. Finally, the remaining 32,482 cases (including 24,502 men [75.4%]; overall average age = 46.6 ± 11.3 years) were included in the statistical analysis (see Fig. 1).

According to the criteria for defining obesity in China, the subjects were divided into four groups by BMI [17]: underweight (BMI < 18.5 kg/m^2^), normal weight (18.5 ≤ BMI < 24.0 kg/m^2^), overweight (24.0 ≤ BMI < 28.0 kg/m^2^), and obese (BMI ≥ 28.0 kg/m^2^). An increase in BMI was associated with higher values for age, SBP at all five physical examinations, heart rate (HR), TC, and salt intake, and being male, drinking alcohol, engaging in physical exercise, smoking, having diabetes mellitus (DM), having hypertension, and taking antihypertensive drugs (*P* < 0.05). As BMI increases, the mean value of ARVSBP gradually increases (*P* < 0.0001). After the underweight group was combined with the normal-weight group (because there were only 490 underweight cases), the mean ARVSBP and the proportion of ARVSBP above or equal to the average increased with an increase in BMI (*P* < 0.0001) (see Table 1).

**Table 1:** Subjects’ basic characteristics by BMI group

Variable	BMI < 18.5 (*N* = 490)	[18.5,24) (*N* = 12,369)	[24,28) (*N* = 13,622)	> = 28 (6001)	Total (*N* = 32,482)	*P*
Age(year)	43.0 ± 14.4	45.8 ± 11.7	47.6 ± 10.9	46.4 ± 11.12	46.6 ± 11.3	< 0.0001
HR(beat/min)	74.4 ± 12.4	73.3 ± 10.0	73.1 ± 9.47	73.9 ± 9.69	73.4 ± 9.78	< 0.0001
TC(mmol/L)	4.66 ± 0.99	4.79 ± 1.08	4.94 ± 1.19	4.98 ± 1.15	4.89 ± 1.14	< 0.0001
SBP1	114 ± 15.7	121 ± 17.5	129 ± 18.5	134 ± 19.2	127 ± 18.9	< 0.0001
SBP2	116 ± 16.9	123 ± 17.8	130 ± 18.1	135 ± 18.6	128 ± 18.7	< 0.0001
SBP3	118 ± 17.6	124 ± 17.3	131 ± 17.7	135 ± 17.9	129 ± 18.1	< 0.0001
SBP4	118 ± 17.7	125 ± 17.8	131 ± 17.9	135 ± 17.7	129 ± 18.3	< 0.0001
SBP5	124 ± 18.0	131 ± 19.3	138 ± 19.3	141 ± 18.9	136 ± 19.7	< 0.0001
Male,n(%)	295 (60.2)	8705 (70.4)	10,768 (79.0)	4734 (78.9)	24,502 (75.4)	< 0.0001
Drinking alcohol, n(%)	64 (13.1)	2103 (17.0)	2475 (18.2)	915 (15.2)	5557 (17.1)	< 0.0001
physical exercise, n(%)	43 (8.8)	1556 (12.6)	1838 (13.5)	791 (13.2)	4228 (13.0)	0.005
Salt intake, n(%)	44 (9.0)	1125 (9.1)	1478 (10.9)	740 (12.3)	3387 (10.4)	< 0.0001
Smoking, n(%)	160 (32.7)	4245 (34.3)	4801 (35.2)	1999 (33.3)	11,205 (34.5)	0.046
Dm, n(%)	20 (4.1)	516 (4.2)	1043 (7.7)	616 (10.3)	2195 (6.8)	< 0.0001
Hypertension, n(%)	55 (11.2)	2949 (23.8)	5489 (40.3)	3205 (53.4)	11,698 (36.0)	< 0.0001
antihypertensive drugs, n(%)	3 (0.6)	471 (3.8)	1149 (8.4)	749 (12.5)	2372 (7.3)	< 0.0001
BPV	11.8 ± 5.82	12.2 ± 6.31	12.8 ± 6.59	13.2 ± 6.85	12.64 ± 6.53	< 0.0001
≥mean BPV	194 (39.6)	4903 (39.6)	5859 (44.0)	2782 (46.4)	13,868 (42.7)	< 0.0001
BPV^a^	–	12.00 ± 6.30	12.8 ± 6.59	13.2 ± 6.85	12.6 ± 6.53	< 0.0001
≥mean BPV^a^	–	5097 (39.6)	5859 (44.0)	2782 (46.4)	13,868 (42.7)	< 0.0001

^a^BPV and BPV ≥ average after merging the underweight (BMI < 18.5 kg/m^2^) and normal-weight (BMI < 24.0 kg/m^2^) groups. *BMI* Body mass index, *HR* Heart rate, *TC* Total Cholesterol, sbp1, sbp2, sbp3, sbp4 and sbp5 Systolic blood pressure of the first to the fifth physical examinations, *DM* Diabetes Mellitus, *BPV* Blood pressure variability

The excluded subjects (*n* = 69,028) were older; had higher SBP, HR, and TC; were more likely to be men, to engage in physical exercise, to take antihypertensive drugs, to have hypertension, and to have DM; and were less likely to smoke, compared with the included subjects (*P* < 0.05). The differences in BMI, salt intake, and drinking alcohol were not statistically significant (see Table 2).

**Table 2:** Basics characteristics of included and excluded subjects in 2006

Gruop	Including people (*N* = 32,482)	Excluding people (*N* = 69,028)	Total (*N* = 101,510)	*P value*
Male, n(%)	24,502 (75.4)	56,608 (82.0)	81,110 (79.9)	< 0.0001
Age, year	46.6 ± 11.3	54.4 ± 12.5	51.9 ± 12.7	< 0.0001
SBP1, mm Hg	127 ± 18.9	133 ± 21.8	131 ± 21.1	< 0.0001
SBP2, mm Hg	128 ± 18.7	135 ± 21.8	132 ± 21.1	< 0.0001
SBP3, mm Hg	129 ± 18.1	134 ± 20.6	131 ± 19.6	< 0.0001
SBP4, mm Hg	129 ± 18.3	135 ± 20.4	132 ± 19.6	< 0.0001
SBP5, mm Hg	136 ± 19.7	141 ± 21.3	138 ± 20.5	< 0.0001
BMI, kg/m^2^	25.1 ± 3.47	25.0 ± 3.51	25.0 ± 3.49	0.432
HR, beat/min	73.4 ± 9.78	74.0 ± 10.4	73.8 ± 10.2	< 0.0001
TC, mmol/L	4.89 ± 1.14	4.98 ± 1.15	4.95 ± 1.15	< 0.0001
Hypertension, n(%)	11,698 (36.0)	32,957 (47.7)	44,655 (44.0)	< 0.0001
Smoke, n(%)	11,205 (34.5)	22,590 (32.7)	33,795 (33.3)	< 0.0001
Salt intake, n(%)	3387 (10.4)	7148 (10.4)	10,535 (10.4)	0.724
Drink, n(%)	5557 (17.1)	12,038 (17.4)	17,595 (17.3)	0.198
Exercise, n(%)	4228 (13.0)	11,053 (16.0)	15,281 (15.1)	< 0.0001
antihypertensive drugs, n(%)	2372 (7.3)	8942 (13.0)	11,314 (11.1)	< 0.0001
Dm, n(%)	2195 (6.8)	7128 (10.3)	9323 (9.2)	< 0.0001

sbp1, sbp2, sbp3, sbp4 and sbp5: Systolic blood pressure of the first to the fifth physical examinations; *BMI*: Body mass index; *HR*: Heart rate; *TC*: Total Cholesterol; *DM*: Diabetes Mellitus

### Stepwise multivariate linear regression analysis of BMI and long-term blood pressure variability

With ARVSBP as the dependent variable and BMI (continuously measured) as the independent variable of interest, the results showed that BMI was positively correlated with ARVSBP. After adjusting for age, gender, drinking alcohol, smoking, engaging in physical exercise, salt intake, TC, HR, DM, and taking antihypertensive drugs, a one-unit increase in BMI was associated with an increase in ARVSBP of 0.077 (*P* < 0.0001) (see Table 3).

**Table 3:** Stepwise multiple linear regression of ARVSBP on continuous BMI^a^

Valueable	B(95%CI)	Beta	*Se*	*P*	Vif
Age	0.14 (0.14–0.15)	0.24	0.003	< 0.0001	1.16
Gender	−0.29(−0.57--0.14)	−0.019	0.086	0.001	1.22
HR	0.026 (0.018–0.033)	0.038	0.004	< 0.0001	1.02
TC	0.13 (0.065–0.19)	0.22	0.032	< 0.0001	1.04
Drinking	0.45 (0.26–0.64)	0.026	0.10	< 0.0001	1.25
physical exercise	−0.35(−0.57--0.14)	−0.018	0.11	0.001	1.08
Dm	0.80 (0.52–1.09)	0.031	0.15	< 0.0001	1.04
BMI	0.077 (0.056–0.097)	0.041	0.01	< 0.0001	1.05
antihypertensive drugs	1.99 (1.71–2.27)	0.24	0.14	< 0.0001	1.08

^a^Adjusting for age, gender, smoking, drinking alcohol, salt intake, physical exercise, TC, HR, DM, and taking antihypertensive drugs. *BMI* Body mass index, *DM* Diabetes Mellitus, *HR* Heart rate, *TC* Total Cholesterol

### Logistic regression analysis of the impact of BMI on long-term blood pressure variability

**Fig. 2:**
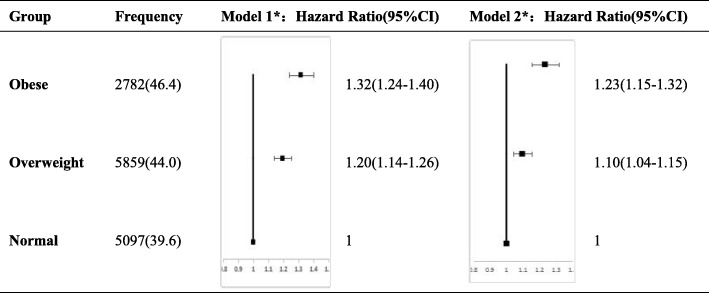
Forest plot of the logistic analysis of factors affecting the increase of ARVSBP; *P for trend = 0.0001; Model 1: unadjusted; Model 2: adjusted for age, gender, smoking, drinking alcohol, salt intake, physical exercise, TC, HR, DM, and taking antihypertensive drugs

In this part of the analysis, ARVSBP group was the dependent variable, and BMI group was the independent variable of interest, with normal-weight as the reference category. Before adjusting for other confounding factors, the ORs showed that the odds of ARVSBP being above average were 1.32 (95% CI: 1.24–1.40) times higher for the obese group and 1.20 (95% CI 1.14–1.26) times higher for the overweight group, compared with the normal-weight group. After adjusting for age, gender, alcohol consumption, smoking, physical exercise, salt intake, TC, HR, DM, and taking antihypertensive drugs, obesity and overweight were still risk factors for an increase in ARVSBP, with ORs for ARVSBP being above average of 1.23 (95% CI: 1.15–1.32) and 1.10 (95% CI: 1.07–1.19), respectively, both of which were statistically significant (Fig. 2).

### Sensitivity analysis

The sensitivity analysis was conducted after separately excluding those with hypertension, those taking antihypertensive drugs, those who were underweight, and measures of BP from the fifth physical examination. Even after adjusting for age, gender, smoking, drinking alcohol, physical exercise, salt intake, TC, HR, DM, and other confounding factors, BMI and ARVSBP were still positively correlated: For every one-unit increase in BMI, ARVSBP increased by 0.027, 0.081, 0.080, or 0.080 in the reduced samples (Table 4).

**Table 4:** Stepwise multivariate linear regression analysis of continuous BMI and ARVSBP among sample subgroups

population	model	B(CI)	Beta	SE	R(R2)	*P*
Excluding person with hypertension	Model a	0.028 (0.005–0.052)	0.016	0.012	0.232 (0.054)	0.019
Excluding person with treatment for BP	Model b	0.081 (0.06–0.10)	0.044	0.011	0.261 (0.068)	< 0.0001
Excluding the BP in 2014	Model c	0.080 (0.059–0.10)	0.038	0.011	0.252 (0.063)	< 0.0001
Excluding person in low-BMI	Model d	0.080 (0.059–0.10)	0.041	0.011	0.284 (0.080)	< 0.0001

Models a and b: adjusting for age, gender, smoking, drinking alcohol, salt intake, physical exercise, TC, HR, and DM. *DM* Diabetes Mellitus, *HR* Heart rate, *TC* Total Cholesterol

Models c and d: adjusting for age, gender, smoking, drinking alcohol, salt intake, physical exercise, TC, HR, DM, and taking antihypertensive drugs. *DM* Diabetes Mellitus, *HR* Heart rate, *TC* Total Cholesterol

## Discussion

This study found that BMI and ARVSBP are positively correlated and that people with higher BMI have higher long-term BPV. After adjusting for DM, TC, and other confounding factors, each one-unit increase in BMI is associated with an increase in ARVSBP of 0.077. No previous studies have examined the association of BMI and long-term BPV. However, in a 15-year follow-up study of 681 children with an average age of 14, Li et al. [18] found that, in an adjusted model, every one-unit increase in BMI was associated with an increase in long-term BPV of 0.029. The results of the present study are consistent with previous studies on the relationship between BMI and short-term BPV. Observing 14,988 people who had undergone health examinations in the United States, Faramawi et al. [8] found that each one-unit increase in BMI was associated with an increase in short-term BPV of 0.25. In their observation of hypertensive people, Qian et al. [19] found that the nighttime SBP among obese people was 20.06% higher than that of the normal-weight group.

The inclusion of subjects who were taking antihypertensive drugs and the use of different measurement methods could have had an impact on our findings for ARVSBP. Thus, the present study included a sensitivity analysis that was conducted separately, excluding those with hypertension, those taking antihypertensive drugs, those in the underweight group, and BP measured at the fifth physical examination. The results of this analysis showed that, for every one-unit increase in BMI, ARVSBP increased by 0.028, 0.081, 0.080, or 0.080 in the models excluding the above-mentioned categories, compared with an increase of 0.077 in the original model. This suggests that BMI may have a stronger impact on ARVSBP among hypertensive patients. Studying 6441 health examination cases over a 6-year follow-up, Cao et al. [20] found that long-term BPV increased in hypertensive patients. In addition, we found that, with the separate exclusion of those taking antihypertensive drugs, those whose BP was measured using a different method, and the underweight group, the effect of BMI on ARVSBP increased. This indicates that antihypertensive drugs weaken the impact of BMI on long-term BPV, there is minimum manual measurement error using an electronic sphygmomanometer, and the lower ARVSBP in the underweight group weakens the effect of BMI on ARVSBP.

This study found that, compared with people of normal weight, the risk of an increase in ARVSBP was 1.23 times higher among obese people and 1.10 times higher among overweight people, after adjusting for diabetes mellitus, antihypertensive drugs, and other confounding factors. This trend was statistically significant. This suggests that the risk of an increase in ARVSBP goes up with a rise in BMI. In addition, there is a dose-dependent relationship between ARVSBP and BMI; that is, the higher the BMI, the higher the ARVSBP. To our knowledge, there has been no previous study of the association between the risk of long-term BPV and obesity.

Because BMI is convenient to measure and widely used in studies related to overweight and obesity, it was selected as the primary indicator of obesity in this study. Previous studies have shown both BMI and waist circumference to be positively correlated with short-term BPV [18]. BMI and waist circumference are also highly correlated with each other; Freedman et al. [21] found a strong correlation between BMI and waist circumference (r ≈ 0.9) in a 12-year follow-up of 32,663 adults.

The long-term regulation of BP is affected by a variety of internal and external environmental factors, such as neuroendocrine factors, and vascular wall elasticity. The present study found that high BMI and ARVSBP were positively correlated. The possible reasons for this correlation are as follows: (i) Sleep apnea syndrome may cause long-term BPV to rise by increasing the synthesis of the angiotensin-converting enzyme, and the incidence of sleep apnea is 1.56 times higher among obese people than among people of normal weight. This increased incidence of sleep apnea among obese people may lead to a higher possibility of increases in long-term BPV [22, 23]. (ii) Vascular wall elasticity is the main regulator of increased BPV. Adipose tissue is an endocrine organ that can release a large number of inflammatory factors, and the long-term damage of inflammatory factors to the endothelial cells leads to higher vascular stiffness, resulting in increased BPV [24, 25]. (iii) The depressor reflex of the baroreflex receptor is an important mechanism for BPV, which is negatively correlated with the sensitivity of the baroreceptor. BPV may be higher among overweight and obese people because the long-term increase of blood volume decreases the vascular baroreflex sensitivity [26].

This study is of public health and clinical significance. Obese people have higher BP and long-term BPV. Clinicians can prevent or control hypertension and reduce long-term BPV by advising obese people to lose weight and advising those of normal weight to avoid obesity. For the stabilization of BP among obese hypertensive individuals, clinicians should consider the patient’s BP fluctuations in addition to his or her BP when using antihypertensive drugs.

There are some limitations to this study. First, the study population was limited to Kailuan Group staff, and the study failed to consider the factor of race. However, the study population was relatively large, so our findings are still important for the study of obesity and long-term BPV in the Chinese population. Second, BP measurements were collected only on the day of the physical examination; there was no collection of family BP or multiple BP measurements during the year. The relatively long follow-up period (average follow-up of 10 years) of the study makes it important for understanding BPV. Third, in accordance with the strict exclusion and inclusion criteria, many individuals were excluded from the study. As a result, higher values were observed for SBP, hypertension, and age among those who were excluded than among those who were included. These differences may have led to an underestimation of the impact of BMI on ARVSBP. Fourth, because this was a prospective cohort study, the association between BMI changes and BPV could not be explored, and there is relatively weak evidence on this association in previous research. The relationship between changes in BMI and long-term BPV requires further study.

## Conclusions

BMI and ARVSBP are positively correlated, with ARVSBP increasing along with a rise in BMI. BMI is a risk factor for an increase in ARVSBP.

## Abbreviations

95%CI: 95% confidence intervals
ARV: Average real variability
AVRSBP: Average real variability of systolic blood pressure
BMI: Body mass index
BP: Blood pressure
BPV: Blood pressure variability
CVEs: Cardiovascular events
DM: Diabetes Mellitus
HR: Heart rate
OR: Odds Ratios
SBP: Systolic blood pressure
TC: Total Cholesterol

## References

[1] Marie N, Tom F, Margaret R (2014). Global, regional, and national prevalence of overweight and obesity in children and adults during 1980–2013: a systematic analysis for the Global Burden of Disease Study 2013. Lancet.

[2] Lim SS, Vos T, Flaxman AD (2013). A comparative risk assessment of burden of disease and injury attributable to 67 risk factors and risk factor clusters in 21 regions, 1990-2010: a systematic analysis for the global burden of disease study 2010. Lancet.

[3] Shihab HM, Meoni LA, Chu AY (2012). Body mass index and risk of incident hypertension over the life course: the Johns Hopkins precursors study. Circulation.

[4] Bombelli M, Facchetti R, Sega R (2011). Impact of body mass index and waist circumference on the long-term risk of diabetes mellitus, hypertension, and cardiac organ damage. Hypertension.

[5] Colangelo LA, THIs V, Szklo M (2015). Is the Association of Hypertension with Cardiovascular Events Stronger among the lean and Normal weight than among the overweight and obese?. Hypertension.

[6] Wilson PW, D'Agostino RB, Sullivan L (2002). Overweight and obesity as determinants of cardiovascular risk: the Framingham experience. Arch Intern Med.

[7] Mirzaei B, Abdi H, Serahati S (2017). Cardiovascular risk in different obesity phenotypes over a decade follow-up: Tehran lipid and glucose study. Atherosclerosis.

[8] Faramawi MF, Fischbach L, Delongchamp R (2015). Obesity is associated with visit-to-visit systolic blood pressure variability in the US adults. J Public Health (Oxf).

[9] Mancia G (2012). Short- and long-term blood pressure variability: present and future. Hypertension.

[10] Murakami S, Otsuka K, Kubo Y (2005). Weekly variation of home and ambulatory blood pressure and relation between arterial stiffness and blood pressure measurements in community-dwelling hypertensives. Clin Exp Hypertens.

[11] Modesti PA, Morabito M, Bertolozzi I (2006). Weather-related changes in 24-hour blood pressure profile: effects of age and implications for hypertension management. Hypertension.

[12] Wu SL, Huang ZR, Yang XC (2012). Prevalence of ideal cardiovascular health and its relationship with the 4-year cardiovascular events in a northern Chinese Industrial City. Circ Cardiovasc Qual Outcomes.

[13] Sega R, Cesana G, Bombelli M (1998). Seasonal variations in home and ambulatory blood pressure in the PAMELA population. Pressione Arteriose Monitorate. E Loro Associazioni J Hypertens.

[14] Zakopoulos NA, Tsivgoulis G, Barlas G (2005). Time rate of blood pressure variation is associated with increased common carotid artery intima-media thickness. Hypertension.

[15] Mena L, Pintos S, Queipo NV (2005). A reliable index for the prognostic significance of blood pressure variability. J Hypertens.

[16] Hastie CE, Jeemon P, Coleman H (2013). Long-term and ultra long-term blood pressure variability during follow-up and mortality in 14 522 patients with hypertension. Hypertension.

[17] Zhou B (2002). Predictive values of body mass index and waist circumference to risk factors of related diseases in Chinese adult population. Zhonghua Liu Xing Bing Xue Za Zhi..

[18] Li Z, Snieder H, Su S (2010). A longitudinal study of blood pressure variability in African-American and European American youth. J Hypertens.

[19] Qian Y, Wang GT, Zhang W (2002). The relation between overload and variability of blood pressure and overweight or obesity in patients with essential hypertension. Zhonghua Liu Xing Bing Xue Za Zhi.

[20] Cao H, Wu S, Li S (2014). Characterization and influencing factors of visit-to-visit blood pressure variability of the population in a northern Chinese industrial city. Chin Med J.

[21] Freedman DS, Ford ES (2015). Are the recent secular increases in the waist circumference of adults independent of changes in BMI?. Am J Clin Nutr.

[22] Martynowicz H, Porębska I, Poręba R (2016). Nocturnal blood pressure variability in patients with obstructive sleep apnea syndrome. Adv Exp Med Biol.

[23] Koyama RG, Esteves AM (2012). Prevalence of and risk factors for obstructive sleep apnea syndrome in Brazilian railroad workers. Sleep Med.

[24] Brunner EJ, Shipley MJ, Oliveira e Silva L (2015). Adiposity, obesity, and arterial aging: longitudinal study of aortic stiffness in the Whitehall II cohort. Hypertension.

[25] Diaz KM, Veerabhadrappa P, Kashem MA (2012). Relationship of visit-to-visit and ambulatory blood pressure variability to vascular function in African Americans. Hypertens Res.

[26] Grigoriadis G, Bunsawat K, Bo F (2016). Blood pressure variability and baroreceptor sensitivity in normotensive obese in response to aerobic exercise. Artery Research.

